# Transcriptome Analysis of Granulosa Cells Reveals Regulatory Mechanisms Related to Chicken Follicle Development

**DOI:** 10.3390/ani14010020

**Published:** 2023-12-20

**Authors:** Xubin Du, Qizhao Zhu, Huifang Pian, Xiaolong Yang, Dong Zhao, Xinyue Wu, Jiawen He, Debing Yu

**Affiliations:** 1 Department of Animal Genetics, Breeding and Reproduction, College of Animal Science and Technology, Nanjing Agricultural University, Nanjing 210095, China; dxb950227@163.com (X.D.); aaronzhu2023@163.com (Q.Z.); 15666289565@163.com (H.P.); wxy18943109916@163.com (X.W.); hjw996640759@163.com (J.H.); 2 Single Molecule Nanometry Laboratory (Sinmolab), Nanjing Agricultural University, Nanjing 210095, China; zhaodong19881206@126.com; 3 College of Animal Science, Xizang Agricultural and Animal Husbandry University, Linzhi 860000, China; 18282942357@163.com

**Keywords:** granulosa cell, follicle development, transcriptome, steroid hormones

## Abstract

**Simple Summary:**

The laying hen industry plays an important role in the production value of the Chinese poultry industry, and the laying performance of hens is usually closely related to their follicle development. Studying the changes in the physicochemical state of the granulosa cells of laying hens during follicle development is helpful to better understand the order of development, which is of great significance in guiding laying hen breeding and production. In this study, transcriptome sequencing technology was used to reveal key regulatory genes in the follicular development of laying hens, and several potential target genes were screened. At the same time, we found that granulosa cell proliferation occurs mainly in the pre-hierarchical follicular period, whereas in the preovulatory period, granulosa cells complete their differentiation and begin to take on complex biological cell functions, including the synthesis of steroid hormones. Furthermore, as pre-hierarchical follicle granulosa cells progress to preovulatory follicle granulosa cells, the genes associated with steroid hormone synthesis increase dramatically, suggesting that steroid hormones play a critical role in follicle development.

**Abstract:**

In this study, we aimed to better understand the difference between the functions of the two types of granulosa cells and sought to discover more key genes involved in follicle development and follicle selection. Herein, we separately collected pre-hierarchical follicle granulosa cells (PHGCs) and preovulatory follicle granulosa cells (POGCs) for RNA extraction; the transcriptomes of the two groups were compared via RNA-seq. A total of 5273 differentially expressed genes (DEGs) were identified between the PHGCs and POGCs; 2797 genes were up-regulated and 2476 were down-regulated in the PHGCs compared with the POGCs. A qPCR analysis confirmed that the expression patterns of 16 randomly selected DEGs were highly consistent with the RNA-seq results. In the POGCs, many of the genes with the most significant increase in expression were related to steroid hormone synthesis. In addition, the genes with the most significant decline in expression, including *AMH* and *WT1*, were related to the inhibition of steroid hormone synthesis. These results suggest that steroid hormones play a key role in follicle development. Furthermore, a Gene Ontology (GO) analysis revealed that these DEGs were mainly involved in the primary metabolic process, the carbohydrate metabolic process, the cellular process, ribosomes, the cytoplasm, and intracellular processes. A Kyoto Encyclopedia of Genes and Genomes (KEGG) enrichment analysis showed that the DEGs were mainly enriched in steroid biosynthesis, the cell cycle, ribosomes, the TGF-beta signaling pathway, focal adhesion, and so on. We also observed the morphology of the follicles at different developmental stages, and the results showed that the thickness of the granular layer of the small yellow follicles (SYFs) decreased significantly with further development. In addition, we also found that the thickness of the granulosa layer of hens over 300 days old was significantly lower than that of 200-day-old hens. In short, these data indicate that the tissue morphology and function of granulosa cells change throughout follicle development.

## 1. Introduction

The egg production performance of poultry depends upon the follicle development of their ovaries: hens with poor egg production usually show poor follicle development [1]. Therefore, understanding the characteristics and molecular mechanisms of follicle development is of great importance for improving egg production performance. Compared with mammals, the follicle development, maturation, and ovulation of the poultry ovary is a continuous process [2]. There are follicles of different sizes in sexually mature chicken ovaries [3], and earlier researchers defined the developmental stages of chicken follicles according to their appearance and diameter [4], which is now widely accepted and used [4,5,6]. When hens lay eggs, the functionally mature ovary contains hundreds of pre-hierarchical follicles, including small white follicles and large white follicles (SWFs and LWFs), small yellow follicles and large yellow follicles (SYFs and LYFs), 5–6 growing preovulatory follicles (sequentially referred to by volume as F6 or F5, F4, F3, F2, and F1), and 2–4 postovulatory follicles (POFs), which are devoid of oocytes [3]. This is due to the atresia of most follicles during the early developmental stage; only after a follicle is selected as a preovulatory follicle can it continue to develop with almost no apoptosis [7]. Each day, one SYF (6–8 mm) is selected to develop into a preovulatory follicle [4].

Granulosa cells and theca cells are the main functional tissues in the follicle wall and play an important role in regulating follicle growth, development, selection, and ovulation [5,6,8,9]. Follicle development and selection are closely related to the function of the follicle-stimulating hormone (FSH) and follicle-stimulating hormone receptor (FSHR) [10,11]. The FSHR is selectively expressed in ovarian tissues (granulosa, theca, and stromal), and the highest expression level is found in 6–8 mm follicles [12]. Although the cells within the theca layer express FSHRs early and promote steroid hormone synthesis, no changes after follicle selection have been observed [13]. However, at the follicle selection stage, granulosa cells begin to express FSHR due to FSH stimulation [14]. All of this suggests that granulosa cells play a crucial role in follicle selection. In addition, there are many factors that influence follicular atresia, but the most direct is granulosa cell apoptosis [15]. Clearly, granulosa cells in the follicle selection phase are a key point in the study of follicle development.

With the development of technology, many new methods have been improved upon and applied to the study of follicle development in poultry [16], especially RNA-seq technology [17,18]. Therefore, in this study, we collected the granulosa cells of pre-hierarchical follicles, and preovulatory follicles were taken as research objects. A transcriptome analysis was carried out to explore the key genes and regulatory mechanisms involved in follicle development and regulation.

## 2. Materials and Methods

### 2.1. Animals, Samples, and Ethics Statement

All laying hens (HY-LINE VARIETY BROWN) in this study were randomly selected from the Qinglongshan Chicken Farm in Nanjing. The hens were reared under the same conditions, kept in separate cages, and had free access to water and feed. They received 14 h of light per day. The vaccination and management procedures were based on the production process of commercial layers. Six healthy hens with similar egg production were randomly selected and killed by cervical dislocation. The peritoneal cavity was then rapidly dissected, and the follicles were removed and placed in 4 °C saline. The granulosa cells of all PHGCs (sequentially demarcated by volume as SWFs, LWFs, SYFs, and LYFs) and POGCs (sequentially identified by volume as F6 or F5, F4, F3, F2, and F1) were collected and stored separately at −80 °C for RNA extraction [19,20], as described by Gilbert et al. [21]. All animal experiments were approved by the Animal Care and Use Committee of Nanjing Agricultural University (Nanjing, China, SYXK-2019-00085).

### 2.2. Paraffin HE Staining

Healthy laying hens at 200, 300, 400, and 500 days old were selected for slaughter. Their SYFs and F6s were separated immediately after the complete removal of the ovaries [21]. All collected follicle samples were fixed directly in 10% (*v*/*v*) neutral formaldehyde. Tissue sections were prepared, deparaffinized, stained with HE, and observed and photographed using light microscopy.

### 2.3. RNA Extraction, cDNA Synthesis and Sequencing

In this study, three samples contained PHGCs from two hens, and three samples contained POGCs from two hens. Total RNA was extracted from each sample using the RNAiso Plus (TaKaRa, Kyoto, Japan), and the RNA was recovered into a 15 μL elution volume using ddH2O containing 0.1% DEPC. In addition, a NanoDrop spectrophotometer (Thermo Fisher Scientific, Waltham, MA, USA) was used to determine the concentration of the RNA, and the Agilent Bioanalyzer 2100 system (Agilent Technologies, Palo Alto, CA, USA) was used to assess the quality of the RNA. The qualified samples were sent to Beijing Allwegene Co., Ltd. (Beijing, China), where the six samples were used to construct a cDNA library following the standard Illumina protocols. In addition, mRNA was enriched from the total RNA using poly-A oligo-attached magnetic beads with an integrity value > 8.0. Double-stranded complementary DNA was synthesized with random hexamer primers and purified using AMPure XP beads. After assessing the quality of the library using the Agilent Bioanalyzer 2100 system, the six libraries were sequenced via pair-end sequencing on the Illumina HiSeq™ 2000 from Beijing Allwegene Co., Ltd. (Beijing, China). To improve the accuracy of the results, each library was sequenced twice.

### 2.4. Identification of Differentially Expressed Genes

First, the quality of the cDNA fragments obtained after sequencing (raw reads) was assessed using the FastQC program (http://www.bioinformatics.babraham.ac.uk/projects/fastqc/ (accessed on 27 February 2023)). A quality control of the raw data was then performed, including the removal of low-quality reads with a quality value (Q) of <20 bases greater than 50% and more than 10% unknown nucleotides. In addition, the Trimmomatic software (version 0.33) was used to remove the raw data, including the adapter, after which the clean reads were mapped to the chicken genome (Gallus_gallus–4.0, ensemble database) using the TopHat2 splice junction mapper. Based on the clean data, differentially expressed genes (DEGs) were measured as the number of fragments per kilobase of the exon model per million mapped reads (FPKM) using the HTSeq software (version 2.0.2). In the subsequent analysis, only genes with FPKM > 1 were analyzed. The DEGs were detected using the R package DEGseq software (version 1.38.0). A corrected *p*-value of 0.05 and a log2 (fold change) of 1 were used as the thresholds for statistically significant differential expression.

### 2.5. Analysis of GO Category and KEGG Pathway

To gain insight into the functional significance of the DEGs in the granulosa cells between the two groups in our study, we arranged their known biological functions and analyzed their pathway enrichment. GO annotation information for all genes was extracted from the Gene Ontology database (http://www.geneontology.org/ (accessed on 2 March 2023)). A GO term enrichment analysis was then performed using the GOseq software (version 1.22), and the DEGs’ GO terms with corrected *p*-values less than 0.05 were selected as being significantly enriched. The association of the DEGs with different pathways was computed with the Kyoto Encyclopedia of Genes and Genomes (KEGG) (http://www.genome.jp/kegg (accessed on 6 March 2023)) using KOBAS (version 2.0), and a corrected *p*-value of 0.05 was used as the threshold for identifying significantly different pathways.

### 2.6. PPI Network

The STRING online website was used for the PPI network interaction analysis in this experiment (version 12.0, https://cn.string-db.org/ (accessed on 4 November 2023)). A total of 200 DEG (|log2(fold change)| > 5) gene sequences were uploaded to STRING for homologous sequence comparison, and the highest-scoring sequences were used for a STRING analysis. The minimum interaction score was set to 0.400 (medium confidence), and the other parameters were left at the default setting. PPI network mapping using Cytoscape software (version 3.10.0).

### 2.7. Quantitative Real-Time PCR (qRT-PCR) Validation

To validate the RNA-seq results, the expression level of several selected up- and down-regulated genes was confirmed via quantitative real-time PCR (qRT-PCR). Total RNA was extracted from the granulosa cells of the PHGCs and POGCs using RNAiso Plus (TaKaRa, Tyoto, Japan). A NanoDrop spectrophotometer (Thermo Fisher Scientific, Waltham, MA, USA) was used to determine the concentration of the RNA. The total RNA (2 µg) was reverse-transcribed using the Primescript RT Master Mix (TaKaRa, Tyoto, Japan). The primers were designed online using Primer3 Input (version 0.4.0, http://bioinfo.ut.ee/primer3-0.4.0/ (accessed on 10 May 2023)) and synthesized by Shanghai Sangon Co., Ltd. (Shanghai, China). The sequences of the primers are listed in Table 1. The RT-PCR reactions were performed using the QuantStudioTM Design and Analysis software V1.4 with the SYBR Green Master Mix. Each reaction was prepared in a total volume of 20 μL, containing 10 μL of the SYBR Green mix, 2 μL of diluted cDNA, and 0.2 μM of each primer. Amplification was carried out with the following cycling parameters: heating at 95 °C for 10 s, 40 cycles of denaturation at 95 °C for 5 s, annealing at 58 °C for 30 s, and extension at 72 °C for 10 s. Each sample was analyzed in triplicate, and negative controls were included in all qPCR runs. The relative expression of each mRNA was calculated using the 2^−ΔΔ Ct^ method [22]. In addition, β-actin was used to normalize the abundance of the target mRNA.

### 2.8. Statistical Analysis

All data are presented as the mean ± SEM. Differences between the groups were determined via a one-way ANOVA followed by Duncan’s test using the SPSS software (SPSS Inc., Chicago, IL, USA) and were considered significant if *p* < 0.05.

## 3. Results

### 3.1. Analysis of the Granulosa Layer Thickness

**Figure 1:**
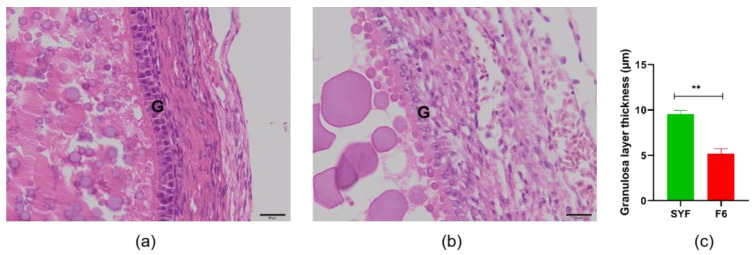
Comparison of SYFs and F6 granulosa layer thickness. (**a**) HE morphology of small yellow follicle. (**b**) HE morphology of F6 follicle. (**c**) Comparison of granulosa layer thickness between small yellow follicle and F6 follicle. Scale bars: 20 μm (40×). G: granulosa layer, *n* = 5, mean ± SEM, ** means the difference is extremely significant (*p* < 0.01).

**Figure 2:**
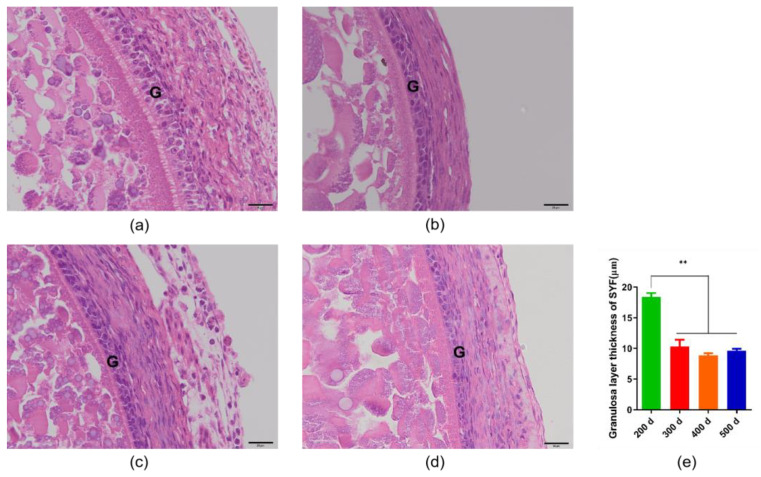
Comparison of granulosa layer thickness of small yellow follicles in hens of different ages. (**a**) HE morphology of small yellow follicle of 200 d layer. (**b**) HE morphology of small yellow follicle of 300 d layer. (**c**) HE morphology of small yellow follicle of 400 d layer. (**d**) HE morphology of small yellow follicle of 500 d layer. (**e**) Comparison of granulosa layer thickness in layers of different ages. Scale bars: 20 μm (40×). G: granulosa layer, *n* = 5, mean ± SEM, ** means the difference is extremely significant (*p* < 0.01).

As shown in Figure 1, we measured the thickness of the granulosa layer of SYFs and F6s. The results showed that, as the follicles developed, the tissue morphology of the granulosa layer of the oocyte also changed, and the thickness of the granulosa layer of the F6s was significantly lower than that of the SYFs. We also tested and compared the thickness of the granulosa layer of SYFs at 200 d, 300 d, 400 d, and 500 d (Figure 2). The results showed that the granulosa layer thickness of SYFs at 200 d was significantly higher than that of other periods.

### 3.2. Summary of Sequencing Results

RNA-seq was used to compare the transcriptomes of three PHGCs and three POGCs, and each sample was sequenced twice to improve the accuracy. A total of 320,048,824 raw reads with a length of 150 bp were obtained from the six samples, and 292,079,676 “clean reads” were gained after eliminating low-quality data via quality control (Table 2). Furthermore, for the six samples, 74.59–76.82% of the clean reads could be mapped to the chicken genome, and more than 93.8% of these reads were uniquely mapped (Table 3).

### 3.3. Identification of Differentially Expressed Genes

**Figure 3:**
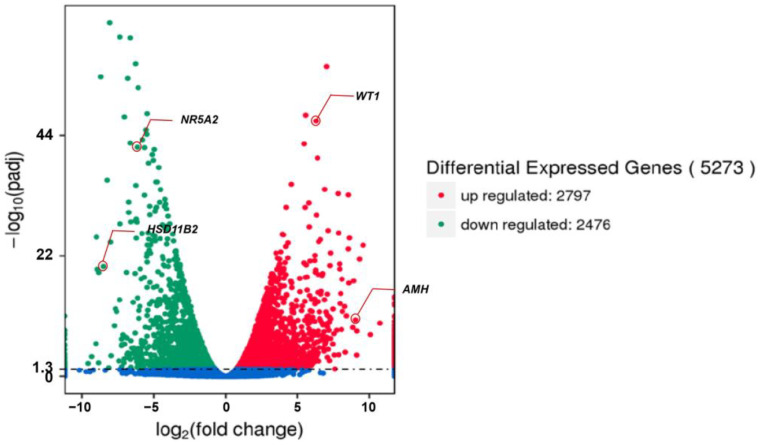
Differently expressed genes between PHGCs and POGCs (PHGCs vs. POGCs). The threshold for DEGs is |log2(fold change)| > 1 and q value < 0.005.

We first examined the global gene expression levels. Based on the FPKM value, it was found that the PHGCs and POGCs had an average of 55.55% and 58.23% genes with a very low expression level (i.e., 0 < FPKM value < 1), respectively. Furthermore, 5.076% and 4.473% of the genes were expressed at a high abundance (i.e., 60 < FPKM value) in the two groups (Table 4). A total of 5273 genes were differentially expressed in the PHGCs and POGCs (Supplementary Table S1). In the PHGCs, the expression level of 2797 genes was significantly up-regulated, and the expression level of 2476 genes was significantly down-regulated (*p* < 0.05) compared with the POGCs (Figure 3). We can observe that, in POGCs, genes involved in the synthesis of steroid hormones, such as *HSD11B2* and *NR5A2*, have the highest fold increase. On the other hand, genes with the highest fold reduction in POGCs, such as *AMH* and *WT1*, have the function of inhibiting the synthesis of steroid hormones.

### 3.4. GO Annotation and Significantly Enriched Analysis of DEGs

**Figure 4:**
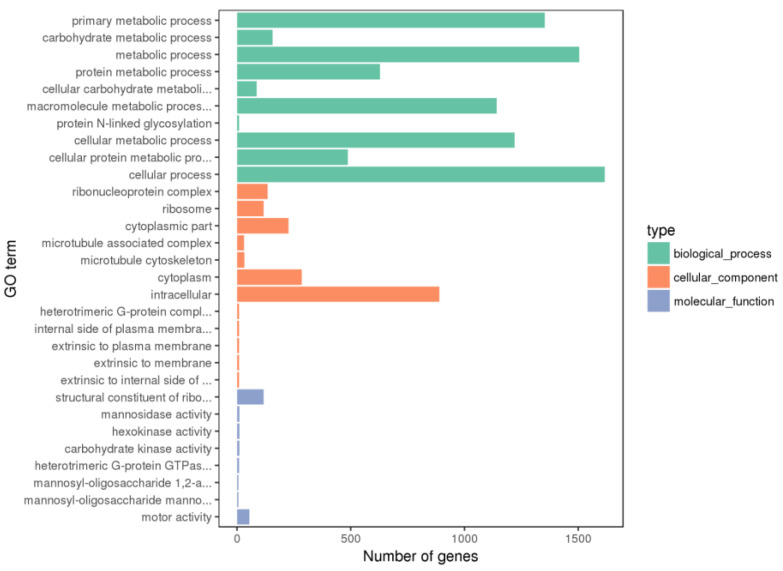
Histogram of GO enrichment. Top 30 GO enrichment terms for DEGs between PHGCs and POGCs. The *x*-axis presents the number of DEGs (both up-regulated and down-regulated). The *y*-axis shows the specific GO term.

The DEGs between the PHGCs and POGCs were annotated, and the functional classification of the top 30 GO terms (including the biological process, cellular component, and molecular function) for all DEGs is shown in Figure 4 and Supplementary Table S2. The DEGs of the PHGCs vs. the POGCs were mainly enriched in the biological process. In the biological process category, the DEGs’ enriched GO terms were classified into the primary metabolic process, carbohydrate metabolic process, etc. In the cellular component category, the enriched GO terms of interest included the ribonucleoprotein complex, ribosome, and cytoplasmic parts. In the molecular function category, the DEGs were enriched in GO terms centered on the structural constituent of ribosomes and mannosidase activity.

### 3.5. KEGG Pathway Enrichment Analysis

**Figure 5:**
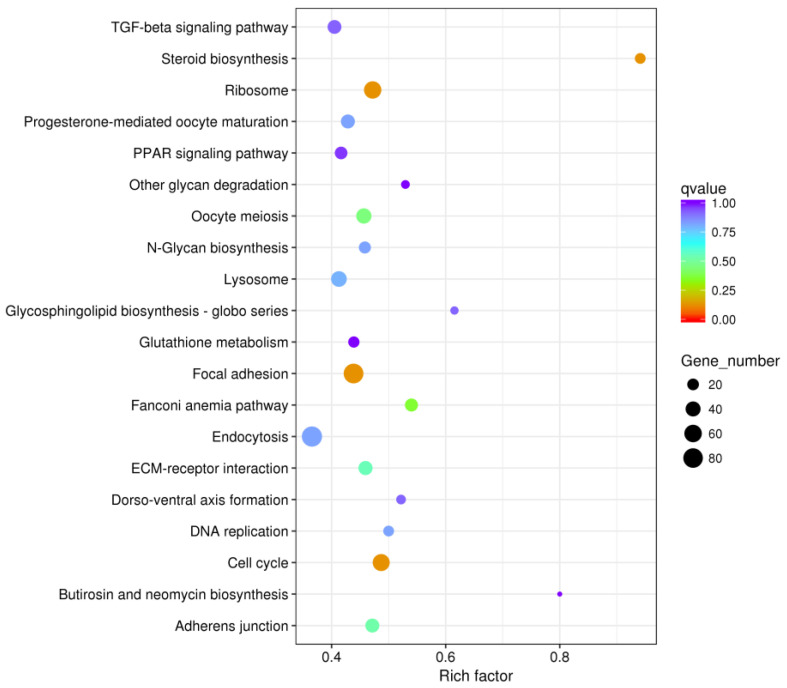
Histogram of GO enrichment. Top 20 GO enrichment terms for DEGs between PHGCs and POGCs. The *x*-axis represents the number of DEGs (both up-regulated and down-regulated). The *y*-axis shows the specific GO term.

The DEGs between the PHGCs and POGCs were assigned to one or more KEGG annotations and were mapped to 152 KEGG pathways. These enriched pathways included steroid biosynthesis, the TGF-beta signaling pathway, steroid biosynthesis, and focal adhesion (Supplementary Table S3). The top 20 data outputs for significant KEGG pathway enrichment are shown in Figure 5. A significant KEGG pathway enrichment of the POGCs compared with the PHGCs was the steroid biosynthesis pathway, with 14 DEGs; however, the largest number of DEGs was in the metabolic pathway (Table 5). Genes in the cell cycle and glycosaminoglycan biosynthesis pathways had higher expression levels in the PHGCs, whereas genes related to the ribosome and steroid biosynthesis pathways were abundantly expressed in the POGCs.

### 3.6. Analysis of PPI Network Results

**Figure 6:**
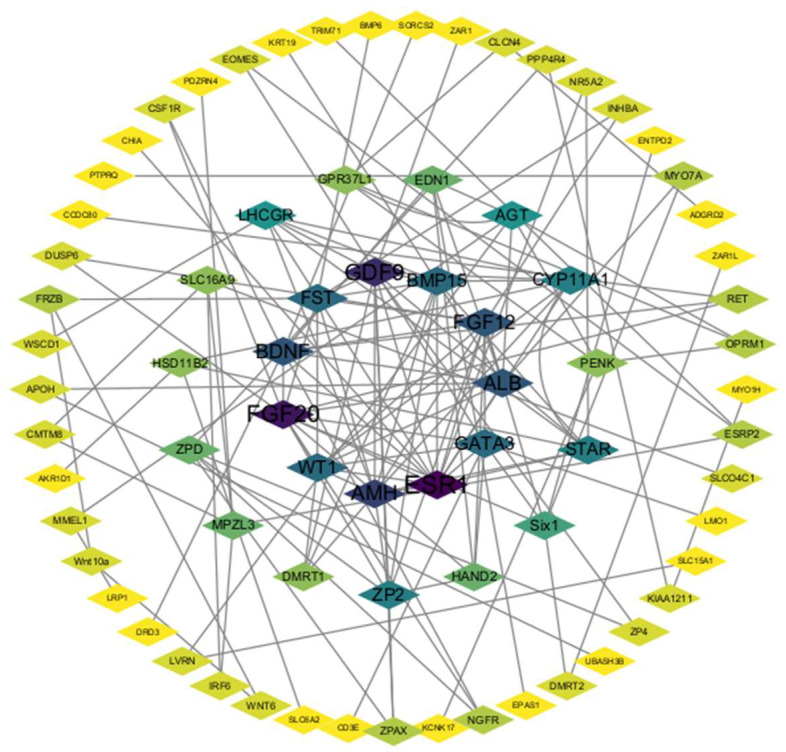
PPI network. Each node represents a gene, and the number of edges between genes represents the number of interacting relationships.

A STRING analysis was performed to investigate the potential interaction network of the DEGs (Figure 6). The results showed that ESR1, WT1, AMH, etc., interacted significantly with each other and connected with many other DEGs, suggesting their role in follicle development. In addition, not all DEGs showed an association with other DEGs because their functions were either unclear or unrelated to each other; these were not included in the analysis.

### 3.7. Confirmation of Selected Genes’ Expression via qPCR

**Figure 7:**
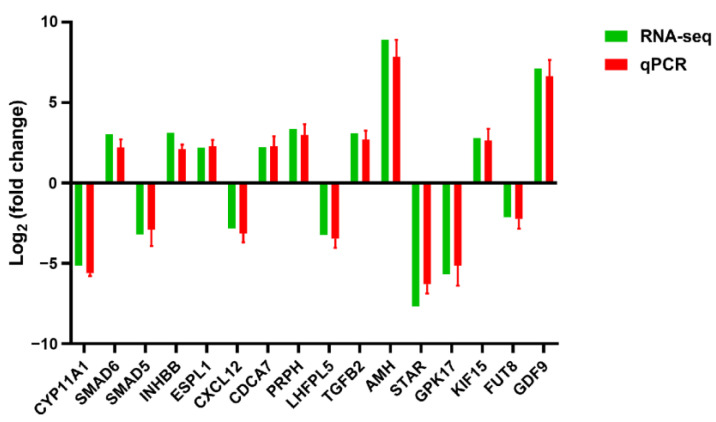
qPCR verifies the results of RNA-seq. There were three samples in total, with three replicates per sample.

Based on the sequencing data and functional analysis, 16 DEGs were randomly selected for validation via qPCR. The results showed that the mRNA levels of these genes were similar to the sequencing data (Figure 7).

## 4. Discussion

The granulosa layer has a different tissue morphology at different stages of development. In this study, we found that the granulosa layer of the SYFs was significantly thicker than that of the F6s. As the primordial follicle recruited from the resting pool grows and differentiates, the flat granulosa cells surrounding the oocyte become square granulosa cells and support the formation of the primary follicle. At this stage, the granulosa cells proliferate and form several layers of somatic cells surrounding the oocyte, allowing the primary follicle to form a pre-hierarchical follicle [23,24,25]. Previous studies have also observed that SWFs have only one layer of cuboidal granulosa cells, whereas the granulosa-layer cells in LWFs, SYFs, and LYFs increase to two to three layers, and the thickness of the granulosa layer and the number of granulosa cells increase significantly with follicle development [26]. In this study, the decrease in F6-thickness GCs may be related to their functional changes; existing research shows that, prior to ovulation, single granulosa cells in a chicken’s follicle within the membrane can be easily transported through the adjacent capillaries alongside nutrients and oxygen, and can act as a systemic hormone to the oocyte [27]. All of this evidence indicates that the morphology of the granulosa layer changes continuously with the stage of follicle development, and these morphological changes may be related to changes in their functions [26]. The low egg production rate of aging laying hens may be related to the decrease in their granulosa layer thickness; studies have shown that the granulosa layer thickness of follicles from 35-week-old hens is significantly higher than that of 75-week-old hens [26]. Our further study compared the granulosa layer thickness of follicles from 200 d to 500 d hens and found that there was no significant difference in the granulosa layer thickness of follicles from 300 d, 400 d, and 500 d hens, whereas the granulosa layer thickness of follicles from 200 d hens was significantly higher than that after 300 d.

The function of chicken granulosa cells changes during follicle development. In this study, we found more than 5000 DEGs between the PHGCs and POGCs. These DEGs are enriched in 150 KEGG pathways. This directly demonstrates that the regulatory network of follicle development is very complex, involving a large number of genes and pathways. It also illustrates the huge difference in function between pre-hierarchical and preovulatory follicles. First, the transcriptome results show that the expression of many genes involved in the cell cycle and DNA replication pathways is significantly higher in PHGCs than in POGCs, indicating that the proliferative capacity of PHGCs is higher than that of POGCs. Previous studies have shown that, in pre-hierarchical follicles, granulosa cells are undifferentiated and have mitotic activity [14]. The multiplication capacity of pre-hierarchical follicle granulosa cells increases as the follicle develops [28]. It has been shown that pre-hierarchical follicles maintain an increasing but undifferentiated growth state, and the MAPK pathway plays an important role in this process [4]. After follicle selection, MAPK signaling is suppressed, and granulosa cells begin to differentiate and undertake various complex biological functions.

There have been many studies on the role of ovarian steroid hormones in follicle development. Steroid hormones play an important role in many processes of follicle development, which requires an appropriate concentration of androgens [29,30,31]. Androgens can either promote [29,32,33] or inhibit [32,34] follicle development by regulating the expression of other genes and also affect follicle activation [35], development [36], and ovulation [37,38]. Estrogen mainly promotes follicle development by inhibiting the apoptosis of granulosa cells [39,40,41,42]. *DHCR7*, *DHCR24*, *HSD3B2*, *HSD11B2*, *HSD17B7*, *CYP2R1*, *CYP51A1*, *LSS*, *SQLE*, *MSMO*, and *NSDHL,* as well as other genes directly involved in the synthesis and transport of steroid hormones, all increase significantly in expression after the development from pre-hierarchical to hierarchical follicles. In particular, *HSD11B2* and *NR5A2* have a higher up-regulation factor. Obviously, these data show that the biggest change in follicles after development from pre-hierarchical follicles to hierarchical follicles is the acquisition of the ability to synthesize steroid hormones. It also demonstrates that ovarian steroid hormones play an important role in regulating follicle development.

Cholesterol is a precursor of steroid hormones, and its deficiency can lead to steroid hormone deficiency [43]. Studies have shown that cholesterol is necessary to activate the classic WNT signaling pathway [44]. DHCR7 and DHCR24 are the key enzymes that convert zymosterol into cholesterol and were significantly up-regulated in this study [45]. A lack of DHCR7 can lead to a reduction in steroid hormones, the depletion of intermediate products, and the accumulation of hydrogen cholesterol (7DHC) [46]. Compared with cholesterol, 7DHC is more prone to oxidation and produces a variety of oxidation products [47,48]. The accumulated 7DHC is not conducive to WNT signaling [49]. Coincidentally, some studies in recent years have found that the WNT signaling pathway plays an important role in the regulation of follicle development [49]. Therefore, in addition to promoting the synthesis of steroid hormones in graded follicles, the influence of DHCR7 and DHCR24 on the development of follicles through the WNT signaling pathway is also worth considering.

In this experiment, we also found that the genes with the most up-regulation in pre-hierarchical follicle granulosa cells are still related to steroid synthesis, although not directly. For example, *AMH* and *WT1* are abundantly expressed in pre-hierarchical follicle granulosa cells, where they inhibit the production of steroid hormones in various ways before they develop into hierarchical follicles. *WT1* has also been shown to inhibit progesterone synthesis in chicken granulosa cells by inhibiting the ERK1/2 signaling pathway and down-regulating the expression of *FSHR*, *STAR,* and *CYP11A1* [50]. *AMH* can inhibit the initial recruitment of primordial follicles and inhibit steroid hormone synthesis and follicle development by reducing follicular sensitivity to FSH [7,51,52]. These data suggest that there is a complex regulatory network at the pre-hierarchical follicle stage that regulates the development of pre-hierarchical follicles into hierarchical follicles from many aspects and also inhibits the synthesis of steroid hormones. They strictly regulate the process of follicle development and maintain the regularity of laying hens.

## 5. Conclusions

The proliferation of granulosa cells mainly occurs prior to follicle selection, and there are multiple mechanisms that inhibit the differentiation of granulosa cells at this stage. After follicle selection, granulosa cells complete differentiation and begin to undertake complex cellular biological functions, including the synthesis of steroid hormones. In short, these data indicate that the tissue morphology and function of granulosa cells change with follicle development.

## Figures and Tables

**Table 1:** Sequences of primers used for qPCR.

Target Genes	Forward/Reversed Primer (5′-3′)	Sequence No.	Product Size
*β-actin*	F: AGTACCCCATTGAACACGGT R: ATACATGGCTGGGGTGTTGA	NM_001206359	196
*CYP11A1*	F: GCTTTGCCTTGGAGTCTGTG R: CGCCATGTCTTGGTGTTGA	NM_001001756.1	181
*SMAD6*	F: CCGATGCCGTGAAGAGGA R: TGTAAGCCCACACACCGTC	NM_204248.2	243
*SMAD5*	F: CAGAAAAGGCCTTCCCCATG R: GAGGTAGAACTGGGCTCTCC	NM_001014968.1	173
*INHBB*	F: AGGGAACCAGAACTTGTTTGTC R: AGCCCTCACATTGAACATCCA	NM_205206.1	266
*ESPL1*	F: CGTTTAACTTTTCCGGGCCA R: CGTTCAAATCTCTCCCGCAG	XM_015273047.2	188
*CXCL12*	F: TTTGCCCTGGCAGTCATCTC R: CACACTTGCTTGCTGTTGCT	NM_001389249.1	182
*CDCA7*	F: TTGCTAACACGAAGCGCAAG R: CGAGGTGGAAACTTCATGGC	XM_025152572.1	232
*PRPH*	F: TTCGCCGCTTTCCTGGAG R: ACGCTTCTGCATCTCATCCT	XM_015272596.2	243
*LHFPL5*	F: GATCGGCGACAGCATCGAC R: GGCACACACTTTGTAGACGG	NM_204398.1	247
*TGFB2*	F: TCATGCGCAAGAGGATCGAG R: TCGGGGTAAAAAGGCTGCAT	NM_001031045.3	237
*STAR*	F: CAACGGAGACAAAGTGCTGA R: AGTGTCCTTCCCAACCCTCT	NM_204686.2	184
*GPR17*	F: TGGCAAAGAGACACACCTCG R: AGTGTCCTTCCCAACCCTCT	XM_001231869.4	247
*KIF15*	F: TTCAAAGCCTGAGCCGAAGA R: AAGCTCCGTGGAATTACACCT	XM_015281734.2	234
*FUT8*	F: CTGGTACGAGACAGTGAGCA R: GTCTTTTCCAAGGCCATCTCC	NM_001004766.1	249
*GDF9*	F: GAAGTGCTGTCTGACCAGGG R: GTCTTTTCCAAGGCCATCTCC	NM_206988.2	185
*AMH*	F: CTGTACCCATCCCTGAGTCC R: AATGGGATCTGCTGTGCTCT	NM_205030.1	174
*WNT6*	F: CAGCCTTCGTGTATGCCATC R: AATGGGATCTGCTGTGCTCT	NM_001007594.2	209

Abbreviations: F: forward primer. R: reversed primer.

**Table 2:** Summary of the RNA-seq results of granulosa cells from pre-hierarchical follicles and preovulatory follicles.

Sample Name	Raw Reads	Clean Reads	Q20	Q30	GC Content
HLPHGC1_1	26,635,294	24,435,125	98.55%	96.13%	51.06%
HLPHGC1_2	26,635,294	24,435,125	96.83%	92.34%	51.07%
HLPHGC2_1	26,842,105	24,329,041	98.61%	96.26%	52.45%
HLPHGC2_2	26,842,105	24,329,041	96.85%	92.42%	52.46%
HLPHGC3_1	26,655,462	24,230,687	98.68%	96.40%	51.73%
HLPHGC3_2	26,655,462	24,230,687	96.84%	92.36%	51.76%
HLPOGC1_1	26,830,769	24,385,501	98.70%	96.40%	53.12%
HLPOGC1_2	26,830,769	24,385,501	96.64%	92.00%	53.11%
HLPOGC2_1	26,665,454	24,434,195	98.58%	96.16%	52.33%
HLPOGC2_2	26,665,454	24,434,195	96.33%	91.39%	52.31%
HLPOGC3_1	26,395,328	24,225,289	98.63%	96.24%	53.53%
HLPOGC3_2	26,395,328	24,225,289	96.46%	91.59%	53.51%

Q20, Q30: the ratio of the number of bases with a Phred quality value greater than 20, 30 to the total number of bases (clean data).

**Table 3:** Result of clean data from six samples mapped to chicken genome.

Sample Name	HLPHGC1	HLPHGC2	HLPHGC3	HLPOGC1	HLPOGC2	HLPOGC3
Total reads	48,870,250	48,658,082	48,461,374	48,771,002	48,868,390	48,450,578
Total mapped	37,543,114 (76.82%)	36,410,695 (74.83%)	36,916,694 (76.18%)	36,378,862 (74.59%)	36,622,466 (74.94%)	36,757,705 (75.87%)
Multiple mapped	1,561,032 (3.19%)	1,461,733 (3%)	1,354,669 (2.8%)	1,570,786 (3.22%)	2,103,784 (4.3%)	2,258,216 (4.66%)
Uniquely mapped	35,982,082 (73.63%)	34,948,962 (71.83%)	35,562,025 (73.38%)	34,808,076 (71.37%)	34,518,682 (70.64%)	34,499,489 (71.21%)
Read-1	18,312,504 (37.47%)	17,792,882 (36.57%)	18,115,458 (37.38%)	17,853,621 (36.61%)	17,744,076 (36.31%)	17,721,519 (36.58%)
Read-2	17,669,578 (36.16%)	17,156,080 (35.26%)	17,446,567 (36%)	16,954,455 (34.76%)	16,774,606 (34.33%)	16,777,970 (34.63%)
Reads map to ‘+’	17,989,629 (36.81%)	17,499,538 (35.96%)	17,798,932 (36.73%)	17,444,307 (35.77%)	17,299,654 (35.4%)	17,313,664 (35.73%)
Reads map to ‘−’	17,992,453 (36.82%)	17,449,424 (35.86%)	17,763,093 (36.65%)	17,363,769 (35.6%)	17,219,028 (35.24%)	17,185,825 (35.47%)
Non-splice reads	27,727,871 (56.74%)	24,589,545 (50.54%)	24,942,750 (51.47%)	21,371,923 (43.82%)	23,455,697 (48%)	21,225,380 (43.81%)
Splice reads	8,254,211 (16.89%)	10,359,417 (21.29%)	10,619,275 (21.91%)	13,436,153 (27.55%)	11,062,985 (22.64%)	13,274,109 (27.4%)

Total reads: the total number of reads after quality control (i.e., the number of clean reads). Total mapped: the total number of reads that can be compared to the reference sequence. Multiple mapped: the number of reads that are compared to multiple positions in the reference sequence. Uniquely mapped: the number of reads at a unique position of the reference sequence. Reads map to ‘+’, Reads map to ‘−’: align the number of reads to the positive and negative strands of the sequence, respectively. Splice reads: align the same read segment to different outside reads. Non-splice reads: the total number of the same read only aligned to one exon.

**Table 4:** Statistics on the number of genes in different expression level intervals.

FPKM Interval	HLPHGC1	HLPHGC2	HLPHGC3	HLPOGC1	HLPOGC2	HLPOGC3
0~1	16,449 (57.60%)	15,603 (54.64%)	15,539 (54.41%)	16,330 (57.18%)	16,288 (57.04%)	17,271 (60.48%)
1~3	2739 (9.59%)	2859 (10.01%)	2703 (9.47%)	2494 (8.73%)	2439 (8.54%)	2683 (9.40%)
3~15	5058 (17.71%)	5043 (17.66%)	5097 (17.85%)	5216 (18.27%)	5010 (17.54%)	4872 (17.06%)
15~60	3108 (10.88%)	3492 (12.23%)	3630 (12.71%)	3230 (11.31%)	3431 (12.01%)	2574 (9.01%)
>60	1203 (4.21%)	1560 (5.46%)	1588 (5.56%)	1287 (4.51%)	1389 (4.86%)	1157 (4.05%)

**Table 5:** Trend of mainly enriched KEGG pathway (PHGCs vs. POGCs).

ID	Description	Count	BgRatio	Gene Name	PHGCs vs. POGCs
gga03010	Ribosome	31	120	*RPL23*/*RPS10*/*RPL27*/*RPL6*/*RPS4X*/*RPS24*/*RPS13*/*RPL4*/*MRPS10*/*RPS3A*/*RPS8*/*RPL15*/*RPL37A*/*RPL37*/*RPS6*/*RPL31*/*RPS11*/*RPLP0*/*RPS16*/*RPL21*/*MRPS6*/*RPL30*/*MRPS14*/*RPL34*/*RPLP2*/*RPL7*/*FAU*/*RPL35*/*RPS27A*/*RPL18A*/*RPS15A*	UP
gga00100	Steroid biosynthesis	14	17	*DHCR7*/*SOAT1*/*CYP2R1*/*LSS*/*NSDHL*/*CYP51A1*/*MSMO1*/*DHCR24*/*LIPML5*/*SQLE*/*SC5D*/*HSD17B7*/*CYP24A1*/*EBP*	UP
gga04350	TGF-beta signaling pathway	32	90	*NEO1*/*SMURF1*/*SMAD5*/*BMPR2*/*TGFB2*/*GREM1*/*ZFYVE9*/*TGFBR2*/*AMHR2*/*BMPR1B*/*BMP6*/*TGIF1*/*FST*/*RGMB*/*SMAD6*/*CHRD*/*SMAD7B*/*TGFB1*/*MYC*/*FMOD*/*INHBA*/*ID1*/*ID2*/*ID3*/*SMAD3*/*SMAD2Z*/*AMH*/*ID4*/*NBL1*/*BAMBI*/*LOC107051468*/*LOC112530287*	UP
gga04510	Focal adhesion	54	189	*ITGB3*/*MYLKSML*/*RAP1A*/*PIK3CD*/*VAV2*/*FN1*/*PTEN*/*RAPGEF1*/*COL6A3*/*MYLK3*/*TNR*/*TNN*/*PPP1CC*/*SHC4*/*LAMA5*/*COL9A3*/*FLNB*/*FLT4*/*PXN*/*PAK5*/*CAPN2*/*PPP1CB*/*VEGFA*/*PIK3R3*/*VEGFC*/*ITGB8*/*SPP1*/*AKT1*/*PDGFB*/*LAMA1*/*PIK3R1*/*THBS4*/*ITGA2*/*GSK3A*/*LAMA4*/*BIRC2*/*VWF*/*PRKCA*/*COL9A2*/*HRAS*/*ITGA9*/*ITGB5*/*PDGFRB*/*COL2A1*/*PTK2*/*MET*/*PRKCB*/*EGFR*/*TNC*/*IGF1R*/*ACTN1*/*BCAR1*/*PPP1R12C*/*PGF*	UP
gga00533	Glycosaminoglycan biosynthesis	9	16	*B4GALT1*/*B3GNT7*/*B3GNT2*/*B4GALT4*/*B4GALT3*/*CHST2*/*FUT8*/*ST3GAL1*/*ST3GAL2L*	DOWN
gga04110	Cell cycle	28	114	*PTTG2*/*CDC45*/*E2F1*/*SKP2*/*CCNB2*/*CDC7*/*TGFB2*/*CDC20*/*MAD2L1*/*WEE2*/*GSK3A*/*TTK*/*CCNA1*/*YWHAG*/*GADD45A*/*CDKN1A*/*GADD45B*/*ANAPC13*/*TGFB1*/*CDK2*/*MYC*/*SMAD3*/*SMAD2Z*/*BUB1B*/*CDC25A*/*ESPL1*/*CHEK1*/*CDC6*	DOWN

## Data Availability

Data are contained within the article and Supplementary Material.

## Supplementary Materials

The following supporting information can be downloaded at https://www.mdpi.com/article/10.3390/ani14010020/s1, Table S1: HLPHGC vs. HLPOGC.DEGs; Table S2: HLPHGC_ vs. _HLPOGC_diff.GO; Table S3: HLPHGC_ vs. _HLPOGC_diff.KEGG.

## References

[1] Kitamura A., Yoshimura Y., Okamoto T. (2002). Changes in the populations of mitotic and apoptotic cells in white follicles during atresia in hens. Poult. Sci..

[2] Li J., Luo W., Huang T., Gong Y. (2019). Growth differentiation factor 9 promotes follicle-stimulating hormone-induced progesterone production in chicken follicular granulosa cells. Gen. Comp. Endocrinol..

[3] Johnson A.L. (2015). Ovarian follicle selection and granulosa cell differentiation. Poult. Sci..

[4] Johnson A.L., Woods D.C. (2009). Dynamics of avian ovarian follicle development: Cellular mechanisms of granulosa cell differentiation. Gen. Comp. Endocrinol..

[5] Onagbesan O., Bruggeman V., Decuypere E. (2009). Intra-ovarian growth factors regulating ovarian function in avian species: A review. Anim. Reprod. Sci..

[6] Nitta H., Mason J.I., Bahr J.M. (1993). Localization of 3 beta-hydroxysteroid dehydrogenase in the chicken ovarian follicle shifts from the theca layer to granulosa layer with follicular maturation. Biol. Reprod..

[7] Johnson P.A. (2012). Follicle Selection in the Avian Ovary. Reprod. Domest. Anim..

[8] Hocking P.M., Gilbert A.B., Walker M., Waddington D. (1987). Ovarian follicular structure of white leghorns fed ad-libitum and dwarf and normal broiler breeders fed ad-libitum or restricted until point of lay. Br. Poult. Sci..

[9] McGee E.A., Hsueh A.J.W. (2000). Initial and cyclic recruitment of ovarian follicles. Endocr. Rev..

[10] Hernandez A.G., Bahr J.M. (2003). Role of FSH and epidermal growth factor (EGF) in the initiation of steroidogenesis in granulosa cells associated with follicular selection in chicken ovaries. Reproduction.

[11] Johnson A.L., Bridgham J.T., Wagner B. (1996). Characterization of a chicken luteinizing hormone receptor (cLH-R) complementary deoxyribonucleic acid, and expression of cLH-R messenger ribonucleic acid in the ovary. Biol. Reprod..

[12] You S., Bridgham J.T., Foster D.N., Johnson A.L. (1996). Characterization of the chicken follicle-stimulating hormone receptor (cFSH-R) complementary deoxyribonucleic acid, and expression of cFSH-R messenger ribonucleic acid in the ovary. Biol. Reprod..

[13] Kowalski K.I., Tilly J.L., Johnson A.L. (1991). Cytochrome P450 side-chain cleavage (P450scc) in the hen ovary. I. Regulation of P450scc messenger RNA levels and steroidogenesis in theca cells of developing follicles. Biol. Reprod..

[14] Tilly J.L., Kowalski K.I., Johnson A.L. (1991). Stage of ovarian follicular development associated with the initiation of steroidogenic competence in avian granulosa cells. Biol. Reprod..

[15] Yao H.H., Volentine K.K., Bahr J.M. (1998). Destruction of the germinal disc region of an immature preovulatory chicken follicle induces atresia and apoptosis. Biol. Reprod..

[16] Zhao D., Leghari I.H., Li J., Mi Y., Zhang C. (2018). Isolation and culture of chicken growing follicles in 2- and 3-dimensional models. Theriogenology.

[17] Tai Y., Yang X., Han D., Xu Z., Cai G., Hao J., Zhang B., Deng X. (2022). Transcriptomic diversification of granulosa cells during follicular development between White Leghorn and Silky Fowl hens. Front. Genet..

[18] Shen M., Li T., Zhang G., Wu P., Chen F., Lou Q., Chen L., Yin X., Zhang T., Wang J. (2019). Dynamic expression and functional analysis of circRNA in granulosa cells during follicular development in chicken. BMC Genom..

[19] Gilbert A.B., Davidson M.F., Wells J.W. (1978). Role of the granulosa cells of the postovulatory follicle of the domestic fowl in oviposition. J. Reprod. Fertil..

[20] Long L., Wu S.G., Yuan F., Zhang H.J., Wang J., Qi G.H. (2017). Effects of dietary octacosanol supplementation on laying performance, egg quality, serum hormone levels, and expression of genes related to the reproductive axis in laying hens. Poult. Sci..

[21] Gilbert A.B., Evans A.J., Perry M.M., Davidson M.H. (1977). A method for separating the granulosa cells, the basal lamina and the theca of the preovulatory ovarian follicle of the domestic fowl (*Gallus domesticus*). J. Reprod. Fertil..

[22] Livak K.J., Schmittgen T.D. (2001). Analysis of Relative Gene Expression Data Using Real-Time Quantitative PCR and the 2^−ΔΔCT^ Method. Methods.

[23] Uyar A., Torrealday S., Seli E. (2013). Cumulus and granulosa cell markers of oocyte and embryo quality. Fertil. Steril..

[24] Etches R.J., Petitte J.N. (1990). Reptilian and avian follicular hierarchies: Models for the study of ovarian development. J. Exp. Zool. Suppl..

[25] Amsterdam A., Hanukoglu I., Suh B.S., Keren-Tal I., Plehn-Dujowich D., Sprengel R., Rennert H., Strauss J.F. (1992). Oncogene-transformed granulosa cells as a model system for the study of steroidogenic processes. J. Steroid Biochem. Mol. Biol..

[26] Hao E.-Y., Wang D.-H., Chen Y.-F., Zhou R.-Y., Chen H., Huang R.-L. (2021). The relationship between the mTOR signaling pathway and ovarian aging in peak-phase and late-phase laying hens. Poult. Sci..

[27] Zhu G., Chen X., Mao Y., Kang L., Ma X., Jiang Y. (2015). Characterization of annexin A2 in chicken follicle development: Evidence for its involvement in angiogenesis. Anim. Reprod. Sci..

[28] Tan T.Q., Ge C., Mi Y., Jin Y., Zhang C. (2010). Ginsenosides promote proliferation of granulosa cells from chicken prehierarchical follicles through PKC activation and up-regulated cyclin gene expression. Cell Biol. Int..

[29] Willis D.S., Watson H., Mason H.D., Galea R., Brincat M., Franks S. (1998). Premature response to luteinizing hormone of granulosa cells from anovulatory women with polycystic ovary syndrome: Relevance to mechanism of anovulation. J. Clin. Endocrinol. Metab..

[30] Takayama K., Fukaya T., Sasano H., Funayama Y., Suzuki T., Takaya R., Wada Y., Yajima A. (1996). Immunohistochemical study of steroidogenesis and cell proliferation in polycystic ovarian syndrome. Hum. Reprod..

[31] Kimura S., Matsumoto T., Matsuyama R., Shiina H., Sato T., Takeyama K., Kato S. (2007). Androgen receptor function in folliculogenesis and its clinical implication in premature ovarian failure. Trends Endocrinol. Metab..

[32] Yang J.-L., Zhang C.-P., Li L., Huang L., Ji S.-Y., Lu C.-L., Fan C.-H., Cai H., Ren Y., Hu Z.-Y. (2010). Testosterone Induces Redistribution of Forkhead Box-3a and Down-Regulation of Growth and Differentiation Factor 9 Messenger Ribonucleic Acid Expression at Early Stage of Mouse Folliculogenesis. Endocrinology.

[33] Sen A., Prizant H., Light A., Biswas A., Hayes E., Lee H.-J., Barad D., Gleicher N., Hammes S.R. (2014). Androgens regulate ovarian follicular development by increasing follicle stimulating hormone receptor and *microRNA*-*125b* expression. Proc. Natl. Acad. Sci. USA.

[34] Kedem A., Fisch B., Garor R., Ben-Zaken A., Gizunterman T., Felz C., Ben-Haroush A., Kravarusic D., Abir R. (2011). Growth differentiating factor 9 (GDF9) and bone morphogenetic protein 15 both activate development of human primordial follicles in vitro, with seemingly more beneficial effects of GDF9. J. Clin. Endocrinol. Metab..

[35] Kezele P., Skinner M.K. (2003). Regulation of ovarian primordial follicle assembly and development by estrogen and progesterone: Endocrine model of follicle assembly. Endocrinology.

[36] Yuan X.-H., Yang C.-R., Wang X.-N., Zhang L.-L., Gao X.-R., Shi Z.-Y. (2019). Progesterone maintains the status of granulosa cells and slows follicle development partly through PGRMC1. J. Cell. Physiol..

[37] Robker R.L., Russell D.L., Espey L.L., Lydon J.P., O’Malley B.W., Richards J.S. (2000). Progesterone-regulated genes in the ovulation process: ADAMTS-1 and cathepsin L proteases. Proc. Natl. Acad. Sci. USA.

[38] Akison L.K., Robertson S.A., Gonzalez M.B., Richards J.S., Smith C.W., Russell D.L., Robker R.L. (2018). Regulation of the ovarian inflammatory response at ovulation by nuclear progesterone receptor. Am. J. Reprod. Immunol..

[39] Salvetti N.R., Ortega H.H., Veiga-Lopez A., Padmanabhan V. (2012). Developmental programming: Impact of prenatal testosterone excess on ovarian cell proliferation and apoptotic factors in sheep. Biol. Reprod..

[40] Britt K.L., Findlay J.K. (2003). Regulation of the phenotype of ovarian somatic cells by estrogen. Mol. Cell. Endocrinol..

[41] Han S.J., Jung S.Y., Wu S.P., Hawkins S.M., Park M.J., Kyo S., Qin J., Lydon J.P., Tsai S.Y., Tsai M.J. (2015). Estrogen Receptor beta Modulates Apoptosis Complexes and the Inflammasome to Drive the Pathogenesis of Endometriosis. Cell.

[42] Chakravarthi V.P., Ghosh S., Dai E., Pathak D., Rumi M.A.K. (2020). Transcriptome datasets of ESR2-regulated genes in rat granulosa cells during gonadotropin-induced follicle maturation. Data Brief.

[43] Cerqueira N.M., Oliveira E.F., Gesto D.S., Santos-Martins D., Moreira C., Moorthy H.N., Ramos M.J., Fernandes P.A. (2016). Cholesterol Biosynthesis: A Mechanistic Overview. Biochemistry.

[44] Sheng R., Kim H., Lee H., Xin Y., Chen Y., Tian W., Cui Y., Choi J.-C., Doh J., Han J.-K. (2014). Cholesterol selectively activates canonical Wnt signalling over non-canonical Wnt signalling. Nat. Commun..

[45] Luu W., Hart-Smith G., Sharpe L.J., Brown A.J. (2015). The terminal enzymes of cholesterol synthesis, DHCR24 and DHCR7, interact physically and functionally. J. Lipid Res..

[46] Prabhu A.V., Luu W., Li D., Sharpe L.J., Brown A.J. (2016). DHCR7: A vital enzyme switch between cholesterol and vitamin D production. Prog. Lipid Res..

[47] Luu W., Sharpe L.J., Capell-Hattam I., Gelissen I.C., Brown A.J. (2016). Oxysterols: Old Tale, New Twists. Annu. Rev. Pharmacol. Toxicol..

[48] Xu L., Davis T.A., Porter N.A. (2009). Rate constants for peroxidation of polyunsaturated fatty acids and sterols in solution and in liposomes. J. Am. Chem. Soc..

[49] Francis K.R., Ton A.N., Xin Y., O’Halloran P.E., Wassif C.A., Malik N., Williams I.M., Cluzeau C.V., Trivedi N.S., Pavan W.J. (2016). Modeling Smith-Lemli-Opitz syndrome with induced pluripotent stem cells reveals a causal role for Wnt/β-catenin defects in neuronal cholesterol synthesis phenotypes. Nat. Med..

[50] Tang S., Li X., Wu X., Gong Y. (2022). WT1 suppresses follicle-stimulating hormone-induced progesterone secretion by regulating ERK1/2 pathway in chicken preovulatory granulosa cells. Gene.

[51] Huang S.J., Purevsuren L., Jin F., Zhang Y.P., Liang C.Y., Zhu M.Q., Wang F., Jia C.L., Wei Z.H. (2021). Effect of anti-mullerian hormone on the development and selection of ovarian follicle in hens. Poult. Sci..

[52] Grossman M.P., Nakajima S.T., Fallat M.E., Siow Y. (2008). Mullerian-inhibiting substance inhibits cytochrome P450 aromatase activity in human granulosa lutein cell culture. Fertil. Steril..

